# Emerging Biomarkers of Multiple Sclerosis in the Blood and the CSF: A Focus on Neurofilaments and Therapeutic Considerations

**DOI:** 10.3390/ijms23063383

**Published:** 2022-03-21

**Authors:** Tamás Biernacki, Zsófia Kokas, Dániel Sandi, Judit Füvesi, Zsanett Fricska-Nagy, Péter Faragó, Tamás Zsigmond Kincses, Péter Klivényi, Krisztina Bencsik, László Vécsei

**Affiliations:** 1 Albert Szent-Györgyi Clinical Centre, Department of Neurology, Faculty of General Medicine, University of Szeged, 6725 Szeged, Hungary; biernacki.tamas@med.u-szeged.hu (T.B.); zsofia.kokas@med.u-szeged.huzsiofia (Z.K.); sandi.daniel@med.u-szeged.hu (D.S.); fuvesi.judit@med.u-szeged.hu (J.F.); fricska-nagy.zsanett@med.u-szeged.hu (Z.F.-N.); farago.peter@med.u-szeged.hu (P.F.); kincses.zsigmond@med.u-szeged.hu (T.Z.K.); klivenyi.peter@med.u-szeged.hu (P.K.); bencsik.krisztina@med.u-szeged.hu (K.B.); 2 Albert Szent-Györgyi Clinical Centre, Department of Radiology, Albert Szent-Györgyi Faculty of Medicine, University of Szeged, 6725 Szeged, Hungary; 3 MTA-SZTE Neuroscience Research Group, University of Szeged, 6725 Szeged, Hungary

**Keywords:** biomarker, diagnostic, prognostic, blood, cerebrospinal fluid, multiple sclerosis, disability progression

## Abstract

Introduction: Multiple Sclerosis (MS) is the most common immune-mediated chronic neurodegenerative disease of the central nervous system (CNS) affecting young people. This is due to the permanent disability, cognitive impairment, and the enormous detrimental impact MS can exert on a patient’s health-related quality of life. It is of great importance to recognise it in time and commence adequate treatment at an early stage. The currently used disease-modifying therapies (DMT) aim to reduce disease activity and thus halt disability development, which in current clinical practice are monitored by clinical and imaging parameters but not by biomarkers found in blood and/or the cerebrospinal fluid (CSF). Both clinical and radiological measures routinely used to monitor disease activity lack information on the fundamental pathophysiological features and mechanisms of MS. Furthermore, they lag behind the disease process itself. By the time a clinical relapse becomes evident or a new lesion appears on the MRI scan, potentially irreversible damage has already occurred in the CNS. In recent years, several biomarkers that previously have been linked to other neurological and immunological diseases have received increased attention in MS. Additionally, other novel, potential biomarkers with prognostic and diagnostic properties have been detected in the CSF and blood of MS patients. Areas covered: In this review, we summarise the most up-to-date knowledge and research conducted on the already known and most promising new biomarker candidates found in the CSF and blood of MS patients. Discussion: the current diagnostic criteria of MS relies on three pillars: MRI imaging, clinical events, and the presence of oligoclonal bands in the CSF (which was reinstated into the diagnostic criteria by the most recent revision). Even though the most recent McDonald criteria made the diagnosis of MS faster than the prior iteration, it is still not an infallible diagnostic toolset, especially at the very early stage of the clinically isolated syndrome. Together with the gold standard MRI and clinical measures, ancillary blood and CSF biomarkers may not just improve diagnostic accuracy and speed but very well may become agents to monitor therapeutic efficacy and make even more personalised treatment in MS a reality in the near future. The major disadvantage of these biomarkers in the past has been the need to obtain CSF to measure them. However, the recent advances in extremely sensitive immunoassays made their measurement possible from peripheral blood even when present only in minuscule concentrations. This should mark the beginning of a new biomarker research and utilisation era in MS.

## 1. Introduction

Multiple Sclerosis (MS) is a common, autoimmune inflammatory and degenerative disease of the central nervous system (CNS) that results in demyelination and the long-term accumulation of disability. It is most prevalent in the northern hemisphere, affects women more than men, and usually manifests in middle-aged patients. Interestingly, both the absolute prevalence and female dominance are reported to have continuously increased in the past several decades [1,2,3]. Even though some symptoms frequently accompany MS, there are no pathognomic symptoms or clinical findings specific to it. Accordingly, MS can present with a wide variety of clinical and imaging changes [4,5]. Before the latest iteration of the McDonald criteria, studies reported that more than half of the patients diagnosed with clinically isolated syndrome (CIS) did not meet the diagnostic criteria for MS. Still, after several years of follow-up, most of these patients eventually did convert to definite MS [6,7]. To overcome this sometimes several year-long lag in the diagnosis, an update was issued to the McDonald criteria in 2017 [8]. The revision still considers MRI as the golden standard in the diagnosis of MS, nonetheless, the reintroduction of oligoclonal bands into the diagnostic toolset also made the diagnostic process easier and much faster. Unfortunately, MRI is not infallible either, as not all demyelinating changes are detected; up to 20% of CIS patients without any characteristic lesions will eventually convert to definite MS [9,10,11]. A growing body of research points towards the multifactorial cause of MS [12]. Even given the exact pathogenesis, several mechanisms and the initiating step in the domino leading to MS are still unclear and opaque, the currently still unfolding interplay between genes and epigenetic regulatory mechanisms, viral [13], environmental, and lifestyle risk factors most certainly play a significant role [12] in it. The principle pathology of MS is characterised by the breakage of the blood-brain barrier, the subsequent infiltration of the CNS by autoreactive lymphocytes followed by demyelination, focal inflammation, and the eventual culmination in axonal loss, gliosis, and white and grey matter atrophy [12,14]. There are three major clinical forms described; most (~85% of all patients) present with relapsing-remitting multiple sclerosis (RRMS), which is characterised by periods of disease worsening (relapses) followed by complete or near-complete resolution of symptoms (remission) with or without persisting residual disability [15,16]. Without treatment after 15–20 years, the reparative and regenerative capacity of the CNS depletes, and the cyclicity of relapses and remissions is superseded by the continuous accumulation of disability, with or without superimposed relapses, termed secondary progressive MS (SPMS) [15,16]. In a minority (~15%) of MS patients, the disease follows a primary progressive (PPMS) course; from the beginning of the disease, continuous progression and disability accumulation are seen, essentially without any relapses or remissive phase [15,16]. In everyday practice, disease activity is monitored by the frequency of relapses (annual relapse rate—ARR), the confirmed disability progression (CDP)—a significant increase of the expanded disability status scale [EDSS] [17] that is sustained for at least three or six months [18,19] and by various MRI parameters (e.g., new or unequivocally enlarging T2 lesions, contrast enhancement, grey matter atrophy) [20]. There are several shortcomings with these clinical measures. Due to the currently used disease-modifying drugs, the ARR of treated patients has fallen so drastically that it became an insensitive marker of disease activity. Also, the specificity and sensitivity of both CDP and ARR are contingent on the frequency of sampling, i.e., the interval between patient visits. Also, most of the accumulating disability in RRMS was shown to be independent of relapse activity, indicating a subtle but continuously present progression [21]. Moreover, the EDSS score is not entirely objective; it is highly dependent on the rater, and shows great inter-rater and intra-rater variability. Furthermore, the patient’s EDSS score may still fluctuate after six months, and sometimes as much as 24 months of follow-up may be necessary for it to stabilize [22,23]. Another pitfall of the currently used disease activity monitoring methods is their temporal displacement. They are unable to forecast future activity by the time they signal that the disease has already progressed (i.e., the damage resulting in the relapse has already occurred, the new lesion seen on the MRI represents inflammation already in progress). The same problem is encountered in everyday practice when a new patient presents with a short disease history. The currently used markers cannot predict future disease activity and the patient’s disease course. Sometimes, it can be extremely challenging to accurately identify a specific disease subtype in the absence of a longer disease course.

In light of this, it is of no surprise that there is an enormous unmet medical need for diagnostic and prognostic biomarkers that can reliably predict a patient’s disease subtype, disease activity and response to treatment before damage is suffered and permanent disability sets in. This might become a reality soon, as recent progress in analytical technologies allows for less invasive and repeat sampling, also enabling the accurate measurement of biomarkers present in the blood, only in minuscule concentrations. Nonetheless, compared with MRI and CSF oligoclonal bands (owing primarily to their lack of specificity and pending validation), these markers have limited contribution to MS diagnosis yet, as such, they are not currently included in the diagnostic criteria. This might change shortly, should the currently seen speed and quality of research continue in the years to come.

Our aim was to compose a narrative review that provides the reader with the most up-to-date information available on the fluid biomarkers of Multiple Sclerosis and their therapeutic implications. In this review, we have included GFAP, leptin, BDNF, copeptin, CH3L1, CXCL-13 and CXCL-11, osteopontin, neurofilament heavy and light chains, micro and circular RNAs, which are the most promising and widely investigated biomarkers. The concise summaries at the end of the sections represent the authors’ concordant professional and personal opinions about the respective biomarkers.

## 2. Fluid Biomarkers of Multiple Sclerosis

### 2.1. Glial Fibrillary Acidic Protein (GFAP)

GFAP is a monomeric, type III intermediate filament protein of 8–9 nm in length coded by the GFAP gene located on the long arm of chromosome 17 in humans [24,25]. It is expressed in various cell types in the body during development. Still, in the central nervous system, it is almost exclusively produced by and found in the cytoplasm of mature astrocytes. Today, the exact role of the GFAP is elusive, but it is primarily thought to play a role in the upkeep of the shape and provide mechanical strength for the astrocytes [26]. Elevated levels of GFAP can be detected in both the blood and CSF after the hyperplasia of the astrocyte population in the CNS. The human brain reacts with astrocytosis and glial scarring to different kinds of insults (trauma, chemical damage, various genetic and non-genetic based disorders, etc.), which results in elevated levels of GFAP in the CSF [27]. Several studies [28,29,30,31,32,33,34,35,36] have demonstrated significant differences in CSF GFAP levels between healthy controls and MS patients, also among different disease subtypes. A most recent meta-analysis [37] has shown a mean difference in CSF GFAP levels of 0.62 (95% CI = 0.56–0.88, *p <* 0.001) between the whole MS cohort and healthy controls (HC). A mean difference of 0.63 (95% CI = 0.39 to 0.86; *p <* 0.001) was observed between RRMS patients and HCs, meanwhile an enormous difference of 103.83 (95% CI = 68.09 to 139.57; *p <* 0.001) was seen between RRMS patients in remission and during a relapse. The mean difference in CSF GFAP levels between progressive MS patients and HCs was 1.02 (95% CI = 0.73 to 1.31; *p <* 0.001), in contrast, progressive MS patients had lower CSF GFAP concentrations than RRMS patients did. (SMD = −0.47; 95% CI = −0.80 to −0.15; *p* = 0.005). There was no difference in CSF GFAP levels between secondary and primary progressive patients (0.35, 95% CI = −0.10 to 0.79; *p* = 0.12). Interestingly, neither natalizumab nor mitoxantrone or rituximab affected CSF GFAP levels [38,39]. Furthermore, CSF GFAP levels have shown a positive correlation with disease duration; this might be explained by the higher degree of astrogliosis accompanying disease progression [40].

There are much fewer studies examining GFAP levels from the blood than research evaluating CSF GFAP levels. A study based on 245 MS patients and 53 controls demonstrated that MS patients had higher blood GFAP levels (difference 37.25, 95% CI = 21.3 to 53.20; *p* < 0.001) compared to HCs [28,32,41]. After differentiation by disease subtype, patients with relapsing-remitting disease were found to have similar blood GFAP levels to those of control subjects (SMD = 0.22; 95% CI = −0.10 to 0.54; *p* = 0.18). In contrast to CSF GFAP levels, PPMS patients had higher serum GFAP concentrations than RRMS patients. Also, serum, but not CSF GFAP levels, correlated with disease severity, especially in patients with a primary progressive disease [28,32,41].

CSF levels of GFAP seem to be correlated with MS and different disease subtypes, reflecting the different extent of damage to astrocytes and subsequent astrogliosis observed in different disease subtypes. As such, GFAP levels may help to differentiate PPMS and RRMS in their early stages, when it is not yet easy to discern the two from each other. Furthermore, GFAP might become a valuable marker of disease severity and progression. Nonetheless, more research with larger cohorts is needed to validate these findings, especially in the case of GFAP measures from the serum.

### 2.2. Leptin

Leptin encoded by the *Ob* [42] gene consists of 167 amino acids and weighs 16 kDa. It is mainly produced by white adipose tissue cells, enterocytes, T-lymphocytes and bone marrow cells [43,44]. Leptin exerts its effect through a type I cytokine receptor [45], plays a pivotal role in regulating several processes such as angiogenesis, wound healing, blood clotting, hunger, energy balance and expenditure, fat storage, hematopoiesis and the immune and inflammatory response of the body [46,47]. In recent years, variations in leptin levels were implicated in the development of MS and other autoimmune diseases [48], as leptin was found to be a key modulator of the immune system [49]. Leptin was shown to have an effect on the neutrophil and macrophage cell lines [50], promote autoreactive T-cell proliferation, inhibit the proliferation of Treg-cells [51], and also to promote the secretion and phosphorylation of several proinflammatory cytokines [52]. The altered expression of these cytokines can push the ratio of Th1 and Th2 regulatory T-cells out of balance, a process associated with the development of MS [53,54,55].

The results of studies exploring circulating leptin levels in MS patients and its potential role as a biomarker and pathogenesis of MS are conflicting. The more significant part of the studies in the literature has found either elevated or no difference in the circulating leptin levels of MS patients compared to healthy controls. On the other hand, some persuasive papers found the exact opposite.

The largest meta-analysis to date in the literature assessing this matter evaluated nine studies, including 645 MS patients and 586 healthy control subjects. Despite a considerable heterogeneity among the articles on which the meta-analysis was based compared to controls, MS patients were found to have significantly higher circulating leptin levels (SMD = 0.70, 95% CI 0.24 to 1.15, *p* < 0.001) [43]. These findings were backed by another study [51], which found that being overweight at the age of 15 and being obese in young adulthood (at the age of 20) increases the risk of developing MS by more than twofold (OR = 2.16, *p* = 0.01 and OR = 3.9, *p* = 0.01, respectively). Similarly, compared to controls, higher leptin levels were seen in RRMS, and even higher levels were measured in SPMS patients. Another adipose tissue-originating cytoplasmic protein, adipocyte fatty acid binding protein (A-FABP), has been markedly elevated in pediatric-onset MS patients compared to control subjects [56]. A Swedish biobank-based study that examined the risk for developing MS based on circulating leptin and insulin levels in patients younger than 40 years corroborated these findings [57]. It found a sex and age-related correlation between leptin levels and the risk of developing MS. Higher leptin levels were associated with increased risk of MS in individuals (both men and women) younger than 20 years (OR = 1.4, 95% CI = 1.1–1.9) and in all evaluated men (OR = 1.4, 95% CI = 1.0–2.0). In contrast, for women aged 30–39 years, there was a lower risk of MS with increased leptin levels (OR = 0.74, 95% CI = 0.54–1.0). The majority of formerly published papers support the argument that elevated leptins are a risk factor for developing MS [56,58,59,60,61,62]. Conversely, a recent study that has assessed different genetic polymorphisms in the leptin and leptin receptor gene has found contradicting results. This Kuwaiti-based study [63] (where obesity is prevalent in the general population) has measured lower leptin levels in MS patients compared to healthy controls. Additionally, they have found that a specific (rs7799039AA) genotype is associated with an elevated risk of developing MS. It was found to bear no effect on the lower leptin levels observed, however.

Unfortunately, most of the studies mentioned above that examined leptin’s association with MS had several serious limitations. Many of the studies had small compound sample sizes, which in many cases diminished to be on the verge of losing statistical power after stratification into the subgroups of interest. Furthermore, most of the articles have failed to correct for several factors known to influence leptin levels (age, sex, smoking status, BMI, treatment status—both disease-modifying drug and steroid administration—of the MS population, disease subtype). Moreover, there is heterogeneity in the sample types that leptin was measured from (serum vs plasma, fasting vs non-fasting sampling). Due to these severe biases, the results and comparability of most of the studies are questionable, at least.

To conclude, there is significant controversy surrounding leptin in MS. Leptin’s suspected contribution to the pathogenesis of MS is based on its ability to modulate the immune system by promoting the production of pro-inflammatory cytokines and recruiting immune cells. Nevertheless, based on currently available data, there is no compelling evidence favouring leptin being a key player in MS pathogenesis, nor for its use as a biomarker in MS.

### 2.3. Brain-Derived Neurotrophic Factor

Brain-derived neurotrophic factor (BDNF) is a member of the neurotrophin family, and due to different splicing, at least 34 different mRNA transcripts are produced in response to different stimuli [64,65,66]. BDNF is widely expressed in the central nervous system and is widely recognised as a major regulator protein for various types of neurons in both the adult and the developing brain [64,67,68]. Via two receptors (the high affinity TrkB and the low-affinity p75 [69]), it plays a vital role in regulating several signalling pathways that control the survival, growth, differentiation and apoptosis of various cell types and is thus pivotal in neural development, neural plasticity and the long-term potentiation of synapses [70,71,72,73]. The most attention and research regarding the genetic variations in the gene coding BDNF has been directed towards the single-nucleotide polymorphism (SNP) rs6265, which causes a valine to methionine substitution at codon 66 (Val66Met). This mutation alters the pro-domain structure of the gene, which is functional; but leads to improper protein folding. This folding failure causes impaired and decreased activity-dependent BDNF release, reduces BDNF binding to its TrkB receptor, and results in altered protein-protein interactions and conformational stability [74]. The Val66Met mutation does not show an even geographical distribution. The Met carriers (Met/Met or Val/Met genotype) make up roughly 1/3rd to half of the Caucasian population across the USA and Europe. Meanwhile, the Met carrier frequency is much higher (~70%) in Asian populations in China, Japan and Korea [75,76,77,78,79,80]. In recent years the Val66Met mutation has been linked to a plethora of psychiatric and neurodegenerative (Huntington’s disease, Parkinson’s disease, Alzheimer’s disease, ALS, MS) diseases. Other polymorphisms of the BDNF gene are also thought to be connected to neuronal disorders [81,82] and impaired visual cognitive processing speed [83]. In contrast, several studies refute the associations above, as they failed to find compelling evidence to link these alterations in the BDNF gene to either Alzheimer’s disease or MS [84,85,86,87,88]. The variance in carrier prevalence across the globe may be at least in part a possible explanation for the various contradicting associations demonstrated between these genetic polymorphisms and neuronal disorders.

Similarly to other neurodegenerative diseases, the prognostic and diagnostic value of BDNF in MS is highly controversial. The Val66Met polymorphism was linked to enhanced grey matter atrophy compared to Val/Val carriers in one study [89], which was refuted by subsequent articles [90,91]. These findings are further shaded by an fMRI-based study, which explored the Val66Met polymorphism’s possible influence on the episodic memory of MS patients. It found that wild type Val/Val carrier RRMS patients compared to Met carrier patients showed greater brain responses during both encoding and retrieval trials on the episodic memory test administered. In contrast, the exact opposite was true for healthy controls. Conversely, a more robust hippocampus-posterior cingulate cortex connectivity was observed in Met carriers compared to Val homozygotes. The exact opposite was true for healthy controls [92]. On the other hand, a most recent study demonstrated that Met carrier status results in low BDNF expression and is a protective factor against cognitive impairment in MS. It has been found to be also associated with worse physical status and to be more prevalent in males [93]. Refuting both previously mentioned papers, a third paper has concluded that BDNF levels show no correlation with the presence of Met/Val polymorphism, or with the patients’ physical status, or any of the psychometric tests used, nor with any of the various MRI parameters measured in the study [94]. To add more to the controversy, in one cohort of MS patients, Val homozygoteousness was associated with younger disease onset in male patients. Meanwhile, it was linked to increased MS susceptibility in females, implying a gender-specific effect of the polymorphism [82]. Contrary to this, several others have failed to show any impact of the BDNF Val66Met polymorphism on the susceptibility, severity, or clinical course of the disease [88,95,96]. As the sole evaluation of the presence or absence of the mutation has resulted in ambiguous results, it was theorised that not merely the polymorphism itself but its epigenetic regulation, namely the methylation status of the BDNF gene, may play a role in the expression and production of BDNF and thus in the pathogenesis and progression of MS. A recent study [97], based on a relatively large Italian cohort assessed this hypothesis and found that merely the presence of rs6265 SNP itself was not a predictor of the severity of the disease. On the other hand, the critical role of epigenetic mechanisms was confirmed: a lower percentage of the methylation of the BDNF gene was associated with a more severe and rapidly progressing disease. Given that higher methylation of a gene results in its silencing, these results suggest that a lower inhibition of the gene (i.e., hypomethylation) results in its hyperexpression (and therefore increased BDNF production in this case) and is associated with a more severe disease course. Bearing in mind that BDNF is considered a neurotrophic factor, it is reasonable to presume that the level of methylation of the BDNF gene is a result of disease activity and not the other way around. It is plausible that patients with a more severe course and a higher level of inflammation resort to de-methylation as a defence mechanism, resulting in a more increased secretion of BDNF, thus suppressing the ongoing inflammation and maintaining as many remaining neural functions as possible. In a similar train of thought, patients with milder disease activity do not need to ease the methylation of the BDNF gene to reduce inflammation in the CNS. Several neuropathological findings support this theory. Both BDNF and its receptor are readily expressed in the vicinity of and in the MS plaques themselves. Furthermore, "burnt out" older and chronic MS plaques seen in later stages of the disease were shown to contain a lesser amount of BDNF [98], which may be one element responsible for the continuously ongoing axonal degeneration in the chronic progressive stage of MS [99,100,101]. Furthermore, evidence indicates that neuronal BDNF might be pivotal to the endogenous neurotrophic repair following axonal damage seen in MS lesions [102,103].

To add to the controversy, there is some substantial inconsistency about the measured blood levels of BDNF in different stages and subtypes of MS. Some have found elevated BDNF in the sera of patients in the relapsing phase [104,105]. In contrast, others have found lower than normal serum levels of BDNF in RRMS patients, regardless of them being in remission or in relapse [106]. Similarly, when compared to HCs, all MS patients (regardless of subtype) in a population level had lower BDNF levels. The lowest concentrations were measured in SPMS patients followed by RRMS patients. This is in line with prior findings that both serum and CSF levels of BDNF are reduced in MS patients compared to HCs [107,108], and that BDNF levels are higher in RRMS than in progressive MS. This further supports the theory that progression in MS may be, in part, at least due to the exhaustion of the endogenous neuroprotective and reparative systems by long-standing chronic inflammation [99,100,101,105,108].

To summarise, BDNF levels and the presence or absence of the Val66Met polymorphism based on current evidence do not seem to be useful biomarkers for predicting disease susceptibility, progression, or for identifying disease subtypes on a patient level. BDNF levels have been relatively concordantly shown in MS patients to be lower than in healthy controls on a population level. The difference, however, is minuscule. Based on current data, it is doubtful that BNDF levels can be of use in any kind of decision-making process regarding either diagnosis or treatment at the patient level. On the other hand, should current results be confirmed by further research, in the future the methylation status of the BDNF gene might provide insight into the predicted disease severity of MS patients.

### 2.4. Copeptin

Copeptin is the more stable C-terminal glycopeptide part of pro-arginine vasopressin, (pro-AVP) consisting of 39 amino acids [109]. It is mainly secreted by the paraventricular and supraoptic nucleus of the hypothalamus and is found in the plasma in equimolar concentration with AVP [110,111]. As it is more stable than AVP, it can be used as a surrogate biomarker [112,113,114]. The hypothalamo-pituitary-adrenal [115] (HPA) axis is initiated and modulated by stressors of different natures [116]. As copeptin is known to be a robust simulator of ACTH secretion, it was suggested to be a potential marker to monitor HPA activity indirectly. Not surprisingly, copeptin has chiefly proven to be a reliable diagnostic marker of cardiovascular diseases [117,118,119] in the pathogenesis of which the dysfunction of the HPA axis and the vasopressinergic system play a pivotal role. In addition, various ante- and postmortem studies have found a disturbance in the HPA system of MS patients [120,121,122,123]. Hyperactivity of the HPA axis has been linked to faster disease progression [120], and enlarged adrenal glands were found postmortem in MS patients, consistent with increased glucocorticoid production [121]. Furthermore, increased levels of cortisol in the CSF [122] and a higher amount of CRH and AVP coexpressing neurons in the hypothalamus have been documented. These alterations were linked to a shorter disease length and faster progression [123]. Moreover, increased cortisol awakening response was shown in EDSS progressor RRMS patients, while cortisol levels in patients with a stable EDDS did not differ from healthy controls [124].

Several studies have demonstrated that copeptin is a viable marker of inflammation and can be used as a prognostic factor for the outcome in different diseases, including those affecting the CNS [116,125,126].

In light of these results, copeptin’s potential value in multiple sclerosis as a biomarker for progression has been raised. Currently, only a few papers have explored this possibility [127,128,129]. Unfortunately, the results of these studies are not compelling. The cohorts examined were diminutive, and every study had a different setup and employed different measurement protocols. Furthermore, there was significant heterogeneity amongst the enrolled patients across the studies; patients with different disease subtypes were recruited, and some studies measured copeptin levels during a relapse. Meanwhile, others examined samples from patients in remission; furthermore, the disease activity, physical disability, comorbidities and other biometric parameters also varied considerably between cohorts. These attributes might have significantly influenced the measured copeptin levels and thus may be responsible for the inconsistent and uncompelling results. Prokopova et al. [129] have evaluated 19 recently diagnosed MS patients in remission before starting their immunomodulatory therapy but after the initial steroid boost for their first relapse. The authors have found mild but prevalent cognitive impairment in the subjects already at the beginning of their disease. Cognitive dysfunction was linked to MS patients (especially in males) having lower BDNF levels than healthy controls. On the other hand, plasma levels of copeptin, cortisol, and aldosterone did not differ between healthy controls and MS patients this early in their disease. This might suggest that the HPA axis hyperactivity—confirmed by other studies—develops later on during the disease. These results have to be taken into account, knowing that patients with obesity or any kind of endocrine disturbance that might influence the HPA axis were excluded from the study [129]. Another study [128] examined 40 newly diagnosed RRMS patients. All participants who were in relapse, therapy-naive, patients who had comorbidities that might have influenced cortisol and/or copeptin levels other than obesity were excluded. The MS cohort as a whole was found to have higher copeptin levels compared to HCs. This difference vanished when only lean patients and control subjects were compared and became more prominent when obese patients and controls were evaluated. Additionally, significantly higher cortisol levels were observed in obese MS patients. When lean and obese MS patients were assessed, no difference was seen in the copeptin levels. Similarly to the control-patient comparison, obese patients were found to have higher cortisol levels than lean patients.

In conclusion, positive correlations were observed between cortisol and copeptin levels in obese MS patients. The results point toward adiposity, not MS itself being the culprit behind the observed alterations of copeptin and cortisol levels in MS patients. The last study addressing the matter [127] enrolled 30 RRMS patients who were in remission for at least a year (MS controls), 19 RRMS patients who have suffered a relapse within one week in whom the copeptin levels were measured during relapse (MS relapse) and reassessed in a month’s time afterwards (MS remission), and 30 healthy controls. Copeptin levels were highest in the MS control group, followed by the MS remission and MS relapse groups. The lowest levels were observed in HCs. No significant correlation was found between the plasma copeptin levels and patient age, disease duration, or EDSS scores. The results of this study suggest the hyperactivation of the HPA axis in MS patients, which is in line with the findings of several previous papers. Several underlying mechanisms and confounding factors influence the HPA axis and influence plasma copeptin levels. Based on current results, copeptin does not seem to be a viable and reliable marker of progression or disease course in MS, especially not on a patient level. Further studies with much larger groups of patients and more uniform inclusion-exclusion criteria and analytical methods are necessary to justify and verify copeptin’s potential role as a biomarker for MS.

### 2.5. Chitinase 3-like 1 Protein (CHI3L1)

Chitinase 3-like 1 protein (CHI3L1) is a glycoprotein secreted by various cell types including, but not limited to, chondrocytes, macrophages, astrocytes, smooth muscle cells, fibroblast-like cells, synovial cells, and activated microglia, to name a few [130,131,132,133,134]. Despite the unknown exact biological function of CHI3L1, it was found to be a key player in inflammation, tissue injury, extracellular tissue remodelling and repair, and fibrosis. This is no surprise, as CHI3L1 was shown to bind to different extracellular matrix constituents and several other molecules regulating the processes mentioned above [130,135,136]. Altered levels of CHI3L1 are strongly associated with many malignant and non-malignant diseases and various neurodegenerative disorders, including MS [130,137,138,139,140,141,142,143]. Multiple factors (age, changes in the extracellular matrix, various cytokines and growth factors, stress, inflammation) have been identified that influence the production of CH3L1, many of which are also prominent factors in the pathogenesis of MS [130]. In the CNS, CHI3L1 is most abundantly associated with astrocytes, activated microglia and macrophages, especially in the regions of inflammation and at sites of reactive gliosis [131].

The first studies exploring CHI3L1 in MS have relied on CSF sampling. In contrast, more recently conducted studies have used blood, making repeat sampling much more accessible and tolerable for the patients. A most recent meta-analysis of the literature [144] has confirmed that CHI3L1’s CSF levels are significantly higher in definitive MS patients than healthy controls (*n* = 486 for MS patients vs. 228 for HCs; the heterogeneity among the studies was insignificant). Several studies also showed CIS patients to have higher CHI3L1 levels compared to HCs, suggesting its overexpression already from the beginning of the disease and highlighting its potential as a prognostic biomarker. Accordingly, the elevation of CHI3L1 in both the CSF and sera of CIS patients was an independent predictor of both disease conversion and more rapid development of disability [145,146,147,148]. On the other hand, there is no significant difference in CHI3L1 levels between RR and progressive subtypes of MS [144]. Similarly, being in remission or in relapse does not influence the CSF levels of RRMS patients [144]. In contrast, higher levels were associated with increased numbers of T2 and Gd+ enhancing lesions on MRI scans and faster disability progression, fulminant disease course [149] and hastened spinal cord atrophy [150,151,152,153,154,155,156]. Furthermore, CHI3L1 proved to be a reliable marker for distinguishing between RR and progressive phenotype and forecasting disability progression when measured together with the neurofilament light chain (NFL) [157]. Natalizumab, fingolimod and mitoxantrone were found to reduce CSF levels of CHI3LI1 in RRMS patients, while for interferon-beta, the same was true only for treatment responders [153,158,159]. On the other hand, glatiramer acetate [156] and dimethyl fumarate (at least in patients with progressive disease [158]) did not influence CHI3L1 levels.

Another chitinase family member, CHI3L2, has also sparked some interest as a potential biomarker in MS. A pilot study [160] has found the predictive capacity of CHI3L2 to be similar to that of CHI3L1. Compared to HCs, elevated levels of CHI3L2 were found in the CSF of patients with optic neuritis; furthermore, patients with higher CHI3L2 levels were more likely to develop MS in the future. Furthermore, CHI3L2 was shown to correlate well with the presence of cognitive impairment [160], and to predict long-term disability progression [161]. Contrasting to CHI3L1, higher CHI3L2 levels at diagnosis were associated with lower baseline EDSS scores in PPMS patients. Furthermore, opposite to CH3L1, lower levels of CHI3L2 were measured in patients with a progressive disease than in RRMS patients [155].

Based on the current data available, both CHI3L1 and CHI3L2 are promising biomarkers in the diagnosis of MS. Furthermore, CHI3L1 seems to correlate well with disease activity and progression in a fashion that DMTs may modify. Strictly speaking, neither CHI3L1 nor CHI3L2 is confined to a specific phenotype of MS. Still, differences in their combined levels might be suggestive of a particular disease subtype. Nonetheless, further confirmatory assessment in larger and more homogenous samples of patients with MS are needed to validate CHI3L1’s and CHI3L2’s status as a biomarker.

### 2.6. C-X-C Motif Chemokine 13 (CXCL13)

CXCL13 is a chemokine protein-ligand of the B-cell receptor CXCR5 [162]. It is one of the most potent B-cell chemoattractants. It is also responsible for the organisation of B-cells within lymphoid follicles [163] and forming ectopic meningeal B-cell follicles and the meningeal tertiary lymphoid organ, which is crucial in intrathecal autoimmunity and the development of MS [164,165]. CXCL13 is expressed in several tissues; not unexpectedly, the highest concentration is seen in organs with lymphoid tissue, such as the spleen, lymph nodes and the gut [166]. As B-cells are known to contribute significantly to the pathogenesis and progression of MS [167,168], it is not surprising that CXCL13 has gained considerable interest as an auspicious biomarker of the humoral immune response in the CNS. Various studies have documented elevated levels of CXCL13 in the CSF of patients with neuroinflammatory diseases, including MS [169,170,171,172,173,174,175].

In the past decade, intrathecal CXCL13 has been established as a valuable prognostic biomarker in CIS patients. Elevated CSF CXCL13 levels are well documented to correlate well with CSF cell count, the presence of oligoclonal bands, and IgG index [176]. Furthermore, high CSF CXCL13 concentration was confirmed by several studies to be associated with an increased risk of conversion to clinically definite MS (CDMS); a higher relapse rate also accurately predicted future disease activity [171,176,177,178]. The use of CSF CXCL13 as a biomarker is not restricted to only CIS patients. In RRMS patients, it was shown to correlate with disease activity and indicators of a more severe disease course, such as the relapse rate, IgG index, intrathecal leukocyte count, cortical atrophy and HLA genotype [169,171,174,179,180,181]. Not surprisingly, CSF CXCL13 levels seem to be a robust and sensitive indicator of intrathecal B-cell response, even under the conditions of an intact blood-brain barrier [167,182]. Accordingly, similarly to RRMS, elevated CSF CXCL13 levels have been associated with disease activity, increased CSF cell counts, IgG-index and MBP, NFL and CHI3L1 concentrations in progressive MS (both PPMS and SPMS) as well [183,184]. Furthermore, as a single marker, the CXCL13 index (calculated as CSF_CXC13_/serum_CXCL13_)/(CSF_albumin_/serum_albumin_) had better specificity, sensitivity, and positive and negative predictive value to forecast future disease activity than OCBs and CSF NFL did. Even higher sensitivity and predictive values were achieved when the CXCL13 index and CSF NFL levels were combined [181]. In addition, elevated levels of intrathecal CXCL13 were detected in 50% of patients treated with highly active DMTs who seemingly had stable disease (no signs of clinical or ongoing radiological activity on MRI), indicating residual, subclinical disease activity [185]. These results further support CXCL13’s greater sensitivity to disease activity than clinical and MRI measures.

The tight correlation between CXCL13 and B-cells and other CSF markers of disease severity makes it a perfect candidate to measure the therapeutic efficacy of the B-cell depleting therapies in MS (ofatumumab, rituximab and ocrelizumab) [186,187,188]. Unfortunately, due to the only recent approval of ofatumumab and ocrelizumab for MS, there are no specific data available yet in the literature regarding these DMTs’ effects on CXCL13 levels, except for rituximab, which is currently used off label for MS [189,190,191,192]. After treatment with rituximab, two chemokines, CXCL13 and CCL19, were shown to significantly decrease in the CSF of patients in correlation with reduced B-cell and T-cell numbers [193,194]. Regarding other MS therapies, CXCL13 has been reported to decrease in patients after treatment with natalizumab or methylprednisolone [152,169,195,196], mitoxantrone [197], but not after interferon-beta treatment [152]. Furthermore, baseline CXCL13 levels were also shown to predict success with fingolimod; nonresponder patients had elevated pretreatment serum levels of CXCL13 compared to patients responsive to fingolimod [198].

Compelling amounts and quality of evidence suggest that CXCL13, especially in CIS patients, is a valuable and reliable prognostic markers of conversion probability and future disease activity. CXCL13 and the CXCL13 index seem to be excellent markers for disease activity and severity in other disease subtypes. Furthermore, if confirmed by studies employing larger cohorts, CXCL13 may have broader utility as a biomarker of therapeutic response, especially in patients on B-cell therapies.

### 2.7. Osteopontin (OPN)

Osteopontin (OPN) is a negatively charged glycoprotein of the extracellular matrix [199,200,201] secreted by a variety of cell and tissue types and several cells of the immune system (including, but not limited to dendritic cells, natural killer cells, T-cells and macrophages). OPN is associated with various physiologic processes (bone mineralisation and remodelling, wound healing, chemotaxis, immune cell activation, apoptosis regulation) and pathologic states including neurodegenerative and inflammatory diseases [201,202,203,204]. On the one hand, OPN looks to be a principal contributor to inflammation resulting in tissue damage. On the other hand, it seems to be a key player in the subsequent reparative mechanisms triggered by the inflammation itself [204,205]. Via several mechanisms and pathways [206,207,208], OPN shifts the cytokine balance towards the proinflammatory side. It increases the production of IL-1β, IL-12, IL-17, IFN-γ and inhibits the expression of IL-10, resulting in detrimental neuroinflammation and the inhibition of lymphocyte death [209,210,211]. In light of this, and as OPN is widely expressed by cells resident in the CNS (virtually on all neurons and glia), and by various immune cells that are either already present in or are capable of migrating into the CNS [208,212], it is not surprising for it to be involved in MS pathology. This theory is supported by the confirmed overexpression of OPN in MS lesions [213], the association of its gene variations with MS susceptibility and progression [214,215], and the plethora of studies reporting elevated levels of OPN in the blood and CSF of MS patients [204,205].

A most recent meta-analysis [204], based on 27 studies, concluded that MS patients irrespective of disease subtype have higher CSF and blood OPN levels than HCs or other patients with non-inflammatory neurological disorders (NIND). When stratified by disease subtype, RRMS patients were found to have the highest CSF OPN levels, followed by CIS and SPMS patients. PPMS patients were shown to have higher CSF and blood OPN levels than CIS patients [204]. Furthermore, higher CSF OPN levels were linked to a higher degree of disability in PPMS [216]. Additionally, concomitantly increased CSF OPN and CXCL12, but not IL-10 levels were measured in PPMS patients compared to RRMS patients [217]. CSF OPN levels were demonstrated to correlate with disease activity; CSF concentration of OPN was shown to elevate in exacerbation of the disease irrespective of disease subtype and subside after the resolution of the attack regardless of whether steroid therapy was administered or not [216]. Accordingly, patients with stable disease had lower CSF OPN levels than patients who showed activity [204]. Despite these alterations in the OPN levels between different MS subtypes, the meta-analysis did not find any difference in the CSF OPN levels of MS patients and patients with other inflammatory neurological diseases [204] No other comparison between MS subtypes has shown a significant difference in blood and/or CSF OPN levels between the examined groups. Another study has found that higher CSF (but not serum) OPN levels at baseline predicted higher white matter lesion volume and white matter loss, increased cortical/subcortical grey matter atrophy, ventricle enlargement and various microstructural alterations in the NAWM of MS patients seen on MRI scans performed more than a decade later [218,219].

Studies in the literature have demonstrated that circulating or CSF osteopontin levels are not specific enough to differentiate MS from other inflammatory diseases affecting the CNS. Moreover, in contrast to the conformity of results on the higher CSF, OPN found in MS patients vs HC results regarding serum OPN levels in MS patients are somewhat conflicting [148,220]. Overall, neither CSF nor serum osteopontin is likely to be useful in the everyday clinical setting as a diagnostic biomarker [221,222]. On the other hand, despite a lack of specificity for MS [223], both CSF and serum osteopontin levels correlate significantly with inflammation, disease activity and clinical severity [216,224,225,226]. Additionally, CSF osteopontin levels might be valuable biomarkers for therapeutic efficacy, as they were shown to respond to treatment with natalizumab and interferon-β [195,227,228,229]. The data from studies suggests that CSF osteopontin, together with other CSF biomarkers of inflammation, may be used to monitor the therapeutic effects on intrathecal inflammation. Nevertheless, additional studies are required to confirm these results with other currently used DMTs and to validate OPN as a disease activity biomarker in the blood.

### 2.8. Neurofilament Proteins

Neurofilaments (Nfs) are presumably the most studied potential biomarker of diseases affecting the nervous system. Nfs are responsible for the cytoskeletal integrity and structure of neurons of the central nervous system (CNS) as well as the peripheral nervous system (PNF) [230]. Nfs are predominantly located in large, myelinated axons of the white matter (WM), but are also present in the grey matter (GM) [231]. Nfs in the PNF neurons constitute NfL, NfM, NfH and peripherin [231], while in the CNS they are composed of NfL, NfM, NfH, and alpha-internexin [232]. Even though their exact function continues to be concealed, they are assumed to play a predominant role in axon stability [233] contributing to adequate nerve conduction velocity [234] and appropriate synapse functioning [235]. Fundamentally, any pathophysiological process or disease resulting in axonal damage can result in increased levels of Nfs. Thus, monitoring changes in Nf levels has a potential relevance as a biomarker in various neurological disorders, as proven by numerous studies [236]. Because of the tissue-specific nature of Nfs, it is comprehensible that these molecules have been the centre of attention in the past three decades [230], and especially studies investigating NfL have demonstrated promising results [237]. NfLs are assumed to enter the CSF either directly as a consequence of neuronal cell membrane disruption, or by active secretion via multivesicular bodies [238]. NfL peptides are thought to enter the bloodstream primarily through intramural periarterial drainage and the glymphatic system [239]. According to various studies, CSF and blood NfL levels show a significant correlation [240,241]. Nonetheless, while CSF NfL levels are principally influenced by the location and the extent of the injury [242], blood NfL levels may also be affected by the integrity of the blood-brain barrier (BBB) [243], PNS damage [244,245], cardiovascular risk factors [246], impaired renal function [247] and increased blood volume [248,249]. Additionally, low concentrations of NfL can be constantly detected in different body fluids, suggesting that NfL is released into CSF and blood as part of physiological processes, such as ageing [247,250,251], ageing-related neurodegeneration and loss of BBB integrity [252,253].

Based on current research, NfL might be a valuable diagnostic [254], differential diagnostic [255], prognostic biomarker [256,257], and also can help to predict the outcome [258,259] and monitor disease activity and therapeutic response [258] in a variety of neurological disorders, including MS.

#### 2.8.1. Neurofilament Light Chain in MS

The first indication that NfL might be a potential body fluid biomarker in MS dates back to 1998. Lycke and colleagues examined the CSF NfL levels of 60 persons with relapsing-remitting MS (pwRRMS) compared to healthy controls over the course of two years. They discovered that individuals with RRMS had significantly elevated NfL levels that correlated with disability and future relapses [260]. And even though NfL is not specific to MS, it has been extensively studied in the past two decades in this field. Several studies evaluated the potential role of NfL use in MS regarding diagnosis, prognosis, disease activity and therapeutic response monitoring.

Initial studies reported elevated levels in MS compared to healthy controls (HCs). Studies reported equal levels of NfL among MS subtypes [261,262], while others found elevated NfL levels in RRMS compared to progressive MS [263,264], suggesting that NfLs get released into the CSF during acute inflammatory activity. In contrast, a few surveys reported that NFL levels were more elevated in PMS compared to RRMS [265,266], indicating that NfL release is also involved in neurodegenerative processes. Elevated NfL levels have been reported with the first demyelinating event in the paediatric population. Furthermore, higher NfL levels were associated with future conversion to RRMS [267] and the early postpartum period [268]. Subsequently, due to the more widespread availability and use of DMTs, sporadic surveys could not identify any difference between NfL levels in MS and HCs [29]. Increased NfL at baseline or during follow-up might represent inflammatory activity, as several investigations declared a correlation with relapses, the occurrence of new T2 hyperintense and Gadolinium enhancing lesion [269,270]. High NfL levels may also reflect progression, since several studies reported an association between high NfL concentration and T1 hypointense lesions and brain atrophy [266,271]. The associations above also raised the possibility that NfL may be suitable to complement MRI examinations, which are currently are the most sensitive in monitoring disease activity and therapeutic response. Implementing NfL measurement into everyday clinical practice as a disease activity and therapy response monitoring tool would involve regular sampling. Even though CSF contains the most considerable amount of NfL and CSF, NfL concentrations are the most sensitive to insults occurring in the CNS [272] because of the invasive nature of lumbar puncture and the fact that NfL eventually enters the circulation via lymphatic drainage or directly as a consequence of BBB disruption associated with a demyelinating event [243]; there has been an increasing emphasis to detect NfL from blood reliably. The first attempts facilitated ELISA [273], while later endeavours exploited electrochemiluminescence (ECL) [270], and in recent years the ultrasensitive single-molecule array (SiMoA) [274,275] became the gold standard technique. The majority of these assessments focused on serum samples [178,270,276,277], while a few utilised plasma [278,279,280], or both [272], to examine the correlation with CSF samples. Despite the different procedures, a significant correlation was ascertained between CSF and serum [261,277,281], as well as CSF and plasma samples [280], and CSF, serum and plasma samples [272]. Other investigations, on the other hand, documented a weak correlation between these sample types [279]. A study compared the three techniques regarding the correlation between CSF and serum samples. SiMoA was revealed to have the highest analytical sensitivity, and CSF and serum samples measured by SiMoA showed the strongest correlation [240]. Another problem with NfL measurements was the low comparability of results due to great inter-facility discrepancies [282]. Sejbeak et al. [282] addressed this topic, and found that NfL levels measured by SiMoA in CSF and plasma in different institutions were comparable, since variability was minor and mainly affected values close to the cut-off. They have also concluded that variability in the laboratory methods might result in more significant discrepancies, making interpretation of values between facilities impractical, thus suggesting the standardisation of measurement methodology.

As previously mentioned, NfL levels correlate with age in HCs. Based on data from 335 healthy individuals, median serum NfL levels are quite similar in the 4th (18.90 pg/mL) and 5th (22.10 pg/mL) decades, but then increase nonlinearly in the 6th (32.4 pg/mL) and 7th decade (43.3 pg/mL). This correlates with mean changes in brain atrophy with a 0.9%, 2.7%, 4.3% and 4.3% change between the ages of 40–50, 50–60, 60–70, >70, respectively, showing stabilisation above a certain age. Interestingly NfL concentration did not correlate with gender [283]. In MS, multiple studies investigated the association between NfL levels and age and gender, with miscellaneous results. Most surveys failed to identify any correlation between CSF and serum NfL and age in MS [261,264,266,272,284]. Some assessments examining NfL levels in MS compared to HCs found no correlation between age and CSF NfL in MS, but strengthened the association mentioned above with age in HCs [274,285,286]. On the other hand, different studies documented a significant relationship between age and CSF, serum, and plasma NfL in MS [40,156,278,287]. The association between NfL and gender is also controversial; most studies found no relationship between CSF and serum NfL and sex [40,271,287,288]. On the other hand, a cohort reported higher serum NfL levels in females [289], while according to other studies, CSF NfL concentrations were more prominent in male patients [290,291].

##### Diagnosis

Numerous surveys assessed the utility of NfL in the diagnosis of MS. Since NfL is not specific to MS, its concentration can rise above normal in other inflammatory neurological diseases and other noninflammatory neurological disorders. Thus, NfL per se is not feasible to diagnose MS [292]. According to the latest diagnostic guidelines, diagnosis of MS can be established if clinical symptoms and/or paraclinical findings support dissemination in time and space [8], and even though the currently used criteria significantly reduced the time previously needed to reach a diagnosis, in individuals with radiologically (RIS) and clinically isolated syndrome (CIS), clinicians can still encounter diagnostic difficulties. Nonetheless, NfL could be a valuable tool to supplement and fasten existing diagnostic processes to reduce the time from first clinical event to diagnosis, and predict future conversion from RIS and CIS to clinically definitive MS (CDMS) discussed below.

A recent study examining 75 RIS patients found elevated CSF NfL levels in RIS converters compared to nonconverters [293], suggesting that higher NfL levels might be a good predictor of future conversion. Another survey investigated CSF NfL and progranulin concentration in an MS cohort comprising RIS, CIS, RRMS, PPMS patients and HCs. It identified elevated NfL levels in CIS, RRMS and PPMS patients. In contrast, only significantly elevated progranulin but not NfL levels were found in RIS patients compared to HCs [294].

Various studies described elevated CSF and serum NfL levels in CIS compared to HCs [274,294,295,296,297] and other non-inflammatory neurological disorders [285]. A recent survey conducted on 177 newly diagnosed CIS and RRMS patients showed increased CSF NfL levels in both groups. There was no statistically significant difference between group NfL levels [298], and this was further supported by others [285]. In contrast, another study described higher CSF NfL levels among RRMS patients compared to patients with CIS [274]. Multiple investigations focused on the predictive value of NfL concerning CIS conversion to CMDS. A prospective study from the Netherlands inspecting paediatric and adult CIS patients reported elevated CSF NfL levels, with higher NfL levels indicative of future conversion to RRMS in both populations [296]. Similarly, a retrospective survey including CIS patients described higher NfL levels among CIS converters compared to nonconverters [270]. In addition, according to a study examining 32 patients with a first demyelinating event, higher baseline CSF NfL levels predicted future relapse and diagnosis of CDMS [299]. CSF NfL levels in combination with other cytokines were also shown to differentiate between isolated optic neuritis and patients later converting to CDMS [178]. Despite convincing results, others refuted these results. Avsar et al., revealed higher GFAP and Tau, but not NfL levels among CIS converters [295]. Distano and colleagues reached a similar verdict. Their results show that more elevated serum NfL levels did not correlate with faster conversion to CDMS [297]. To add to the controversy, a retrospective survey from the US conducted on 120 military persons diagnosed with MS found that serum NfL levels of these individuals were elevated years before diagnosis, and a within-person increase was associated with a shorter time to clinical onset [300]. Ultimately, in case of uncertainty, measurement of NfL might be utilised as a complementary examination to distinguish patients at risk of developing CDMS from non-converters. In addition to identifying CIS patients with a risk of future conversion, higher baseline CSF NfL is associated with disease severity [263], future EDSS progression and conversion to SPMS [288,290]. Data in the literature suggests that not baseline [263] serum NfL but the change in its level indicates future conversion to SPMS [301]. All in all, evidence suggests that baseline and repeat NfL measurement might complement current clinical and paraclinical evaluations to identify patients at risk of conversion. According to a 15-year longitudinal follow-up study, baseline serum NfL levels above 7.62 pg/mL can predict future conversion to SPMS with a 93.3% sensitivity and 46.1% specificity [302].

Another diagnostic dilemma not entirely resolved by routine clinical and MRI examinations is accurately establishing PML risk in JC positive patients receiving natalizumab (NAT). A study investigating 96 patients who received NAT showed that even though serum NfL levels at therapy initiation were similar in patients who later developed PML and patients who did not, NfL levels were reduced in both groups during treatment. In contrast, when measured later in the second year, patients who later developed PML had significantly higher NfL levels than the rest of the cohort [303]. This is further supported by a case report of two patients with PML who showed continuous elevation of serum NfL levels at PML onset, which increased further in a patient having IRIS; however, after treatment, parallel to subsiding PML, serum NfL levels decreased as well [304]. Thus, NfL might be a helpful candidate in monitoring patients at risk of PML and diagnosing PML.

##### Monitoring Disease Activity

As mentioned above, according to numerous studies, elevated NfL levels are associated with clinical and radiological disease activity. At the same time, low concentrations comparable to HCs indicate a stable disease. Moreover, longitudinal studies suggest that NfL levels measured at baseline and throughout follow-up predict future changes. These attributes make NfL a good candidate for monitoring disease activity.

##### Relapse Activity

Countless studies revealed that CSF NfL levels were associated with relapses, implying that axonal damage was most pronounced in the presence of disease activity [152,185,263,305]. High baseline serum NfL levels were shown to correlate with the relapse activity of the previous year [266,298] and to forecast a relapse in the near future (up to 90 days), but not in the subsequent years after sampling [306]. High baseline levels are also associated with the number of future relapses as well [299,307]. Not just flat levels, but elevating kinetic of NfL can also indicate future clinical and radiological disease activity [308,309]. CSF NfL levels remaining high after one year of DMT treatment were shown to convey relapse risk and to predict therapeutic ineffectivity [157]. In contrast to these results, others examining CSF NfL [284] and serum NfL concentration [310] failed to establish any correlation between baseline NfL levels and ongoing [311] or future relapse activity [312].

##### Disability: EDSS, Timed 25 Feet Walk Test (T25FWT) and 9-Hole Peg Test (9HPT)

According to cross-sectional studies, high baseline CSF NfL levels correlate with high baseline EDSS [156,285] and future EDSS progression [276,288,290]. Somewhat contradictory to this data are the results of other studies that confirmed the correlation between baseline CSF [298], serum [289] and plasma [313] NfL levels and baseline EDSS but failed to link them to future EDSS and disability progression. Some studies documented a link between baseline serum NfL and baseline EDSS [243,269,289,297,311], while others did not find such a correlation [314,315,316]. In contrast, a few investigations could not reveal any association at all between CSF [40,157,266,317] or blood [272] NfL levels and EDSS. Interestingly, another study found no correlation between baseline serum NfL levels and baseline EDSS. Interestingly, high baseline serum NfL concentrations predicted high EDSS levels at follow-up and at study end [318]. Not just flat concentrations, but the change in serum NfL levels has also been associated with a change in EDSS [306,310] scores. Furthermore, falling CSF NfL levels after DMT initiation are linked to stabilising EDSS scores [287].

In SPMS, higher serum NfL was linked to hastened whole-brain atrophy, more new/enlarging T2 lesions, and higher T2 lesion volume. In contrast, NfH levels showed no correlation with any clinical or MRI measures [319]. A retrospective study evaluated the serum and CSF NfL concentrations of a population participating in an interferon-beta treatment study has found that both CSF and serum NfL levels taken at years two, three, and four were predictive of patients reaching EDSS 6 at eight and 15 years of follow-up [276].

Data are limited regarding the association between NfL concentration and disability measured by the Timed 25 Feet Walk Test (T25FWT) and 9-Hole Peg Test (9HPT). Some found no correlation between serum baseline NfL levels and baseline 9HPT [310] or T25FWT at baseline [310] or after ten years of follow-up [320]. Meanwhile, others have found that higher baseline serum NfL correlated well with baseline physical disability measured by 9HPT and worse performance in the T25FWT at follow-up [319].

##### MRI Activity

T1, T2 and Gadolinium Enhancing Lesions

As mentioned earlier, several studies demonstrated a correlation between NfL levels and evidence of radiological activity. Baseline CSF NfL levels are associated with baseline [285], and future Gd+ lesions [299]. Similarly, CSF NfL levels were shown to correlate not just with the presence of Gd+ lesions [185,291,321], but with the number of Gd+ lesions [288,322] as well. In contrast, another study found a correlation only between CSF NfL levels and the presence, but not the number of Gd+ lesions [157]. Studies examining serum NfL levels also described an association between baseline serum NfL levels and baseline [243,269,270,276], and future Gd+ lesions [323]. Moreover, an increase in serum NfL levels was associated with the appearance of new Gd+ lesions [309,310,324]. Baseline NfL levels were shown to correlate with the presence and volume of baseline Gd+ lesions; interestingly, no such association was found with Gd+ lesions at 52 weeks [315]. Similarly, others found no correlation between serum NfL levels and the number of Gd+ lesions [271,325].

Several papers evaluated the association between NfL levels and the presence, number and volume of T2 lesions, with mixed results. CSF NfL levels were shown to correlate with both baseline and future [276] T2 lesion load and to predict new T2 lesions as well [272,274,276]. Similarly to CSF, serum NfL levels also correlated with the presence and number of T2 lesions [243,267,306]; vice versa, T2 lesion volume showed correlation with baseline serum NfL concentrations [271,319]. Furthermore, some have showed that the rise of serum NfL levels can predict the progression of T2 lesion load months in advance [309]; meanwhile, others failed to confirm these results [310]. This might be explained by the findings of another study which similarly found no correlation between serum NfL and T1, T2 lesion volumes, measured by conventional MRI. However, high serum NfL levels correlated with T2 lesion volume and normal-appearing white matter (NAWM) damage measured by DTI [311]. Similar results are available regarding T1 lesions; CSF NfL levels correlate with the presence [296] and volume [266] of T1 hypointense lesions. Similarly, serum NfL levels correlate with T1 lesion count [267], volume [269] and future accrual of lesions [271].

##### Brain Atrophy

There is a rapidly rising amount of data assessing the connection between blood/CSF NfL levels and brain atrophy. Not surprisingly, a strong correlation between high CSF NfL concentrations and brain atrophy has been established by several studies [274,276]. Furthermore, one study demonstrated a correlation between CSF NfL levels and grey matter (GM) atrophy [156]. At the same time, another study found that CSF NfL levels to correlate only with the thalamus and nucleus accumbens volumes, but not the whole brain, white matter (WM), GM or putamen atrophy [326]. Nonetheless, in many cases, serum NfL levels were associated with baseline and future brain atrophy [243,271,306,319]. Accordingly, both baseline serum NfL levels and subsequent changes in NfL levels were shown to predict changes in brain volume [327]. This is somewhat contradicted by the findings of Kuhle et al., who have documented that only baseline serum NfL levels, but not the changes in NfL levels, correlate with brain atrophy [310].

##### Cognitive Impairment

As the knowledge regarding neuropsychological symptoms in MS expands, the clinicians’ objective has shifted from solely diagnosing cognitive impairment, fatigue or depression to monitoring and treating these symptoms. The measurement and evaluation of neuropsychological symptoms are based on neuropsychological batteries and patients reported outcomes. It has been suggested that a correlation between NfL levels and neuropsychological symptoms might exist. Accordingly, high serum NfL levels and cortical thickness have been reported to correlate with global neuropsychological performance as measured by the Brief Repeatable Battery of Neuropsychological Tests (BRB-N) and the Paced Auditory Serial Addition Test (PASAT) [325]. In line with these results, a study on 39 patients showed an inverse association between CSF NfL levels and Brief International Cognitive Assessment for MS (BICAMS) test results in progressive but not RRMS [284]. Confirming these findings, CSF NfL levels were shown by another study to correlate with not just overall cognitive impairment but impairments in information processing speed and verbal fluency as well in newly diagnosed CIS and RRMS patients [328]. In contrast, others found no correlation between CSF NfL levels and cognitive impairment measured by the BICAMS battery [321,329] (a weak correlation was observed with the California Verbal Learning Test [CVLT-II] part of the BICAMS test [329]). Interestingly, an increase in serum NfL levels was associated with worse performance on parts of the BICAMS test, but a better score on the PASAT [310]. This might be explained by the generally high PASAT scores (50–60 points) reported at both baseline and throughout follow-up [310]. This is supported by another study demonstrating a weak correlation between baseline serum NfL levels and future PASAT scores [316].

Data are scarce regarding fatigue, depression, and quality of life, but a study reported an association between serum NfL levels at one year and fatigue scores worsening at ten years [320]. Yet another study assessing 38 newly diagnosed CIS and RRMS patients reported no association between serum NfL levels and fatigue [330]. Similarly, another study found no correlation between CSF NfL levels and anxiety or depression [331]. In contrast, a significant correlation was made between baseline serum NfL levels and baseline quality of life measured by the Multiple Sclerosis Quality of Life-54 (MSQoL-54) questionnaire; moreover, serum NfL levels at baseline and follow-up correlated with changes in MSQoL-54’s physical role limitations and social functioning composite scores [289].

##### Monitoring Therapeutic Response

Another area where regular NfL measurement could be utilised is therapeutic response monitoring. As of today, the most sensitive method for this purpose is an MRI examination performed annually or, in case of relapse, emergently. As mentioned earlier, on the one hand, the MRI device itself is expensive and a scarce resource, so it might not be universally available. On the other hand, the examinations are costly; thus, performing regular annual examinations without the occurrence of new or worsening symptoms might not be financed. In this case, another similarly sensitive method for monitoring therapy response may be helpful. Furthermore, disease activity may not always manifest in clinical relapse or radiological changes. In some cases, an increase in NfL levels might be the only sign of disease progression in a patient otherwise showing no disease activity [185]. In such situations, complementing MRI examination with regular NfL measurement may provide additional information to make a therapeutic decision. Thus, the dynamics of NfL concentrations during disease-modifying treatment have been extensively studied to understand better how NfL measurements can be implemented in clinical practice to monitor therapeutic response.

##### Moderately Effective DMTs

Evaluation of patients originally participating in the phase 3 IFN-β clinical trial and its extension studies reported significantly reduced CSF NfL levels in patients treated with IFN-β compared to placebo. Furthermore, an increase in NfL levels among patients treated with IFN-β was associated with a suboptimal treatment response [276]. Another study examined 32 treatment-naïve RRMS patients initiating either glatiramer acetate (GA) or INF-β. After treatment started, first decreasing and then afterwards consistently low NfL levels were documented in therapy-responsive patients. In contrast, NfL levels remained high in nonresponders and correlated with MRI and relapse activity [316].

The effect of dimethyl-fumarate (DMF) on CSF, serum, and plasma NfL levels was assessed in a cohort of 104 previously treatment-naïve RRMS patients receiving either treatment or placebo. At baseline, RRMS patients had higher NfL levels than HCs. After one year of treatment, CSF, plasma, and serum NfL levels had all been reduced to levels comparable to that measured in HCs [272]. Even though a tight correlation was observed between sample types, CSF NfL levels proved most sensitive to relapse and MRI activity [272]. Another trial conducted with DMF employed 54 PPMS patients (27 on DMF, 27 on placebo). CSF NfL levels were reported to be elevated in both arms, but no clinically significant difference in mean NfL change was seen at the end of the follow-up [332]. A pilot study investigating delayed-release DMF in SPMS patients found that CSF NfL levels correlated better with clinical improvements experienced by patients than MRI [333].

##### Highly Effective DMTs

Several studies evaluated NfL in patients receiving natalizumab (NAT). An already significant decrease in CSF NfL concentrations was demonstrated after just 12 months of treatment with NAT in RRMS patients [39,334]. Compared to interferon-β, patients receiving NAT experienced a more significant reduction in CSF NfL levels, supporting previous results that NAT is more effective in preventing axonal damage than moderately effective therapies [152]. Another study reported similar results; CSF NfL levels were significantly reduced in the whole study population receiving NAT for 12 months. Not surprisingly, a surge in NfL levels was observed in the event of a relapse. At the same time, NfL concentrations remained stable in patients with stable disease, further underlining NfL’s potential in monitoring therapeutic response [335]. Moreover, according to the ASCEND phase III study, NAT was able to decrease serum NfL levels in patients with active progressive MS as well [336]. Additionally, according to a small cohort conducted on 11 NAT-treated patients during and after pregnancy, CSF and serum NfL levels during pregnancy remained as low as preconception concentration. However, in the early postpartum period, a transient NfL peak was observed that did not correlate with relapse or MRI or clinical activity, suggesting that NAT was effective in preventing disease activity even in pregnancy [268]. As mentioned previously, serum NfL levels during NAT treatment also correlated with PML risk [303] and furthermore showed a significant increase in the case of PML onset [337]. Thus, NfL levels might not only aid monitoring therapeutic response but may efficiently augment the decision of NAT cessation in JC positive individuals at risk of developing PML.

Different doses of fingolimod (FG) also proved effective in decreasing CSF NfL levels. The change correlated with MRI parameters and relapse rates as well [338]. A study examined patients who either escalated to FG from first-line treatments or switched laterally from NAT. In the case of escalation, CSF NfL showed a significant reduction; in contrast, NfL levels remained persistently low in patients switching from NAT. This suggests that NfL might not only play a role in monitoring treatment response but also in informing about treatment efficacy [159]. A similarly significant decrease in NfL levels after escalation from injectable therapies to FG was also demonstrated in another study [339]. The reduction in NfL levels also correlated with patients’ multiple sclerosis severity scale (MSSS) score. Fingolimod also successfully decreased plasma NfL levels of PPMS patients compared to placebo [340].

A most recent study evaluated the effectiveness of subcutaneous cladribine (CLA), and reported significantly reduced CSF NfL levels at follow-up in a subpopulation of patients with elevated baseline NfL levels [341]. A case report examining patients with progressive MS receiving cladribine reinforced these findings; CSF NfL levels were reduced at follow-up [342], suggesting that cladribine might be a potential candidate in the treatment of PMS showing disease activity and that NfL levels might reflect therapeutic response in PMS as well.

According to a study examining serum NfL levels of RRMS populations participating in the CARE-MS and extension studies, compared to baseline measures, alemtuzumab significantly reduced NfL levels at two years, which was sustained at seven years. Moreover, NfL levels at the end of the observation period were significantly lower in alemtuzumab-treated patients compared to patients receiving IFN-β [269]. Furthermore, in a small cohort of 15 highly active MS patients, elevated baseline serum NfL levels continuously decreased until reaching a stable state after treatment with alemtuzumab. The low NfL levels were coupled with a drop in disease activity measured by the annualised relapse rate and MRI parameters. Accordingly, low NfL levels correlated with no evidence of disease activity, whereas an increase in NfL levels was associated with progression of T2 lesion load and the appearance of new Gd enhancing lesions [309]. In a large cohort, compared to DMF, FG, NAT, teriflunomide and rituximab, alemtuzumab was associated with the lowest on-treatment plasma NfL levels and the highest reduction in NfL levels compared to baseline [343].

The ORATORIO phase 3 randomised clinical trial showed that ocrelizumab effectively decreased the rate of disability progression and serum NfL levels compared to placebo [344]. Unfortunately, real-world clinical data on ocrelizumab are scarce, and future observational studies are needed to confirm these results.

The EXPAND Phase 3 study examined the efficacy of siponimod in SPMS patients. It revealed that after 21 months of follow-up, serum NfL levels in patients receiving treatment were notably decreased, while serum NfL levels in patients in the placebo arm were even increased compared to baseline [345].

In the ASCLEPIOS clinical trial, ofatumumab significantly reduced serum NfL levels at follow-up compared to baseline; meanwhile, the comparator, teriflunomide, only showed a modest decrease in serum NfL levels. Moreover, ofatumumab appeared to be superior to teriflunomide in disability and MRI related endpoints as well [346].

##### Other Therapies

Elevated baseline CSF NfL levels were reported in a cohort of 46 patients receiving autologous haematopoetic stem-cell transplantation (aHSCT). CSF NfL levels decreased significantly after aHSCT [307]. Another study that included 23 patients with aggressive MS supported this finding. After HSCT, CSF and serum NfL levels significantly decreased and remained low in patients who responded to therapy but have remained high in nonreponders [271].

Similarly, a significant decrease in CSF NfL levels was observed in patients who switched to rituximab from either IFN-β or glatiramer acetate. The drop in NfL levels also correlated with MRI measures [347]. Daclizumab was also shown to significantly decrease the number of new contrast-enhancing lesions and CSF NfL levels [348]. In contrast to the previously mentioned DMTs, treatment with ibudilast had no significant effect on the baseline and endpoint CSF and serum NfL levels between individuals receiving therapy and placebo [349]. Similarly, no evidence of therapeutic effect concerning NfL concentration was observed in SPMS patients receiving simvastatin or placebo [319].

A longitudinal follow-up study described an inverse dose-dependent association between serum 25(OH)D vitamin and CSF NfL levels [322]. Consequently, a few studies aimed to examine the outcome of vitamin D supplementation in MS. A randomised clinical trial investigating the effect of vitamin-D compared to placebo found no difference in serum NfL levels between the two groups at the end of follow-up [314,315]. A similar study examining 40 interferon treated RRMS patients receiving high dose vitamin-D or placebo established no significant difference between groups at the end of follow-up. Baseline plasma NfL levels were already low, probably due to interferon treatment. At the end of the follow-up, no significant decrease was observed between the placebo and high dose vitamin D groups [350]. However, the potential effect of vitamin D on NfL levels might have been masked by interferon therapy.

Despite the abundance of studies overwhelmingly indicating that changes in NfL levels represent and correlate well with therapeutic response, there is still only limited evidence on replicating these findings in clinical practice. A recent investigation conducted on 203 patients explored the utility of CSF NfL in therapeutic decision- making [291]. The authors reported that NfL levels were particularly useful in progressive MS, whereas often an NfL increase was the only indicator of ongoing disease activity. This might serve as a warning that in clinical practice, where clinicians mainly rely on clinical symptoms and MRI measures the insidious presence of disease activity may be missed. Taking this into account, NfL might be exceedingly feasible in monitoring the therapeutic response in progressive MS. However, in order for NfL measurement to be part of everyday clinical practice, validation studies, age and concomitant disease-related normal ranges and standardisation of laboratory methodologies are mandatory.

#### 2.8.2. Neurofilament Heavy Chain in MS

Compared to NFL, the role of NfH in MS clinical practise is limited. A study examining CSF NfH levels in CIS compared to non-inflammatory neuropsychiatric disorders revealed significantly elevated NfH concentrations in persons with CIS, which correlated with physical disability and brain volume during the one-year follow-up, further supporting the presence of axonal damage even in the earliest stages [351]. Similarly to NfL, the increased concentration of NfH and tau was shown to be a predictor of conversion from CIS to CDMS [352]. In contrast to NfL, only CSF but not serum NfH levels correlated with EDSS scores [277]. Interestingly, despite a higher increase observed in CSF NfH than in NfL levels during disease activity, NfL still proved to be a more reliable marker for disease stability [335]. Higher NfH levels were also associated with relapse activity [335,353], EDSS [354] and MSSS [355] scores, and T2 lesion volume [355]. These findings are refuted by other studies which failed to demonstrate a correlation between NfH levels and clinical and MRI variables of MS patients [310,319].

The post-mortem examination of brain samples revealed that tissue concentration of hyperphosphorylated NfH shows a strong correlation with NAWM and T1 lesions on post-mortem MRIs [356]. Moreover, serum NfH concentrations were shown to be moderately associated with T2 lesion volumes but not T2 lesion numbers. Conversely, a correlation with the number of T1 hypointense lesions but not with T1 hypointense lesion volume is documented; interestingly, NfH seems to be not related to brain atrophy either [277]. Both CSF NfH and NfL levels were documented to decrease in patients receiving natalizumab with a more pronounced reduction seen in NfL levels [335].

### 2.9. MicroRNA (miRNA)

MicroRNA (miRNA) is a minuscule, single-stranded, hairpin-like piece of non-coding RNA composed of twenty-some (~19–24) nucleotides [357] that is abundantly produced by many cell types. Most of the mapped genes in the human body seem to be targets of miRNAs [358,359]. MiRNAs seem to play a pivotal role in the regulation of several biological processes, which is supported by their exceptional evolutionary conservation [360]. MiRNAs work by regulating gene expression at the post-transcriptional level. They exert their regulatory function by base-pairing with their respective complementary sequence on the targeted mRNA strand. This, in turn, results in the silencing of the affected mRNA molecule by one or more of several processes (i.e., cleavage of the mRNA, decreased translation by ribosomes, loss or shortening of the polyA tail, etc.) [361]. Slightly more than half of the known miRNAs are found within the genes they regulate. Roughly 40% of the mapped miRNA genes lie in the exons or introns of neighbouring host genes in which they are usually regulated together with [362,363,364]. In addition to their abundant intracellular expression, miRNAs are readily secreted into the extracellular compartments (ECmiRNA) as well. They can be found circulating in the body fluids, such as the CSF and blood [365], where they are an integral part of intercellular communication [366]. To date, there is no compelling evidence to determine whether circulating miRNAs are released into the circulation as a cellular byproduct or are specifically released on purpose for a regulatory function. Nonetheless, in contrast to other RNA species, extracellular miRNA molecules are extremely stable [367]. This attribute makes miRNA a perfect candidate for becoming a non-invasive, reproducible and sensitive biomarker for several diseases. Indeed, much attention has been drawn towards miRNAs as biomarkers in the past few years. Not surprisingly, altered miRNA profiles have been discovered in several neurological and autoimmune diseases, including MS [368,369,370,371].

Unfortunately, there is great heterogeneity among studies evaluating miRNAs’ exact role in MS. Most studies have examined the miRNA profiles of peripheral blood mononuclear cells (PBMC) (for an excellent and extensive review see [372]) and different subsets of T- and B-cells of MS patients. In contrast, only limited information is available on the relationship between ECmiRNAs and the miRNA profile of lesions and the normal appearing white matter of MS patients [373,374,375,376]. Despite the different tissue types examined, the common observation of the reports is that of a heavily dysregulated miRNA profile in patients, which points toward a global role for miRNAs in MS pathology.

In spite of great diversity in MS lesion pathology, the studies examining them have found conserved miRNA profiles clustered around inflammation, gliosis, demyelination and remyelination. Most of the aberrantly expressed miRNAs were shown to regulate resident cells of the CNS implied in MS pathophysiology [373,374,375,376,377]. The miRNA profiling of active lesions has shown dysregulation of several miRNAs (some up-, some downregulated) compared to NAWM. The targets of most (miR-155, miR-326, miR-34a, miR-146a, miR-219 and miR-388) of the abnormally regulated miRNAs were found to be responsible for T-cell differentiation, remyelination and macrophage phagocytosis, processes which are known to be prominent in both the development and the resolution of MS lesions [373,378]. An increased amount of miR-326 was reported in both active lesions (most intensely in the Marburg variant of MS) and the peripheral blood of MS patients, particularly during a relapse. The increased production of miR-326 enhances Th-17 cell production and reduces the expression of CD47 (an antiphagocytic signal for macrophages). Both these changes have been suggested to play a role in the pathogenesis of MS [373,379]. Conversely, other miRNAs (miR-126-3p, miR-146b-5p, miR-155, miR-196a-5p, miR-21-5p, miR-223-3p, miR-326 and miR-379-5p) have been shown to be elevated during remission, some of which (miR-223-3p and miR-379) occur only in men [380].

In a most recent meta-analysis, Zailaie et al. [381] aimed to collate research data and summarise the current knowledge of different miRNAs measured in the serum as a diagnostic biomarker for MS. Some miRNAs act as a signature or as a panel, while others as a standalone marker were shown to have high enough diagnostic accuracy in differentiating disease forms from each other and distinguishing between healthy controls and MS patients. Of the several hundred miRNAs profiled, a total of 19 were identified to be significantly differentially expressed in MS patients. Eleven were found to be downregulated (miR-145, miR-376 c-3p, miR-128-3p, miR-191-5p, miR-26a-5p, miR-320a, miR 486-5p, miR-320b, miR-25-3p, miR-24-3p, miR-140-3p), while seven (miR-572, miR-15b, miR-23a, let-7 c-5p, miR-16, miR-24, miR-137, miR-181) were shown to be upregulated in the sera of MS patients compared to healthy controls. The expression level of miR-223 (the 19th identified miRNA) was discordant between the reporting studies [381]. The elevation of five miRNAs (miR-572, miR-15b, miR-223, miR-128-3p, miR-191-5p) was reliably linked to primary progressive disease. Furthermore, several miRNAs were shown to be nonidentically expressed in different disease subtypes; some (miR-128-3p, miR-191-3p, miR-191-5p, miR-24-3p, miR-26a-5p, miR-376 c-3p) were found to be upregulated, while miR-572 was downregulated in the sera of both PP and SPMS patients. MiR-223 and miR-15b were downregulated in PPMS in comparison to SPMS. Conversely, miR-376c-3p was only elevated in PPMS but not in other disease forms. The most dysregulated miRNA in PPMS was found to be circulating miR-191-5p. In contrast, miR-27a-3p was overexpressed in RRMS compared to the progressive forms. Some miRNAs (miR-572, miR-145, miR223, miR-137, miR-16, miR-181, miR-24) were established to have better than average sensitivity and specificity to discern between MS patients and HCs, of which the single best miRNA to distinguish between HCs and MS patients was miR-145, with a sensitivity and specificity of 79% and 87%, respectively [381].

The target identification of the dysbalanced miRNAs’ might explain why the expression of these specific miRNAs is altered in MS. Cytotoxic T-lymphocyte-associated protein 4 (CTLA-4) has been reported to be a target of miR-145 [382]. CTLA-4 is a known inhibitory protein that regulates Treg cell function and enhances the immune system’s ability to suppress auto-reactive T-cells, the overactivity of which, among others, have been implicated in the pathogenesis of MS [383]. The exact mechanism by which the aberration of miRNA expression alters the immune system and its connection to autoimmune diseases is controversial and elusive. A plausible scenario in MS is that the observed upregulation of miR-145 silences the CTLA-4 gene, leading to impaired Treg cell function and the increased proliferation of autoreactive T-cells. MiR-191-5p, despite being abundant in the circulation, was found to be downregulated in the NAWM of PPMS patients [384,385]. BDNF, SOX4, FZD5 and WSB1, genes all implicated in CNS homeostasis, were all found to be targets of and reported to show an inverse correlation with the levels of miR-191-5p in NAWM of MS patients. SOX4 is known to promote neural differentiation in the adult CNS. The verexpression of SOX4 has been shown to inhibit oligodendrocyte differentiation from precursor cells, whereas its downregulation results in increased glial maturation [386]. The intense, progressive neurological dysfunction, axonal damage and neurodegeneration seen in the primary progressive phenotype may be, in part, explained by the observed downregulation of miR-191-5p in the NAWM of PPMS patients. This dysbalance might be one of the causes behind the upregulation of SOX4, which in turn results in impaired oligodendrocyte differentiation and, thus, the failure of remyelination and ultimately in the death of neurons. These findings may explain, in part, at least why MS patients’ CNS is more susceptible to inflammation and less capable of repair.

The relative sparsity of CNS and immune-system related genes, especially in adolescents, proven to be associated with MS, might raise the question that epigenetic changes may play a pivotal role in the pathogenesis of pediatric-onset MS (pedMS). To explore this option, a large scale miRNA genome-wide association study (MIGWAS) and an miR-SNP analysis were conducted on 486 pedMS patients and 1362 control subjects [387]. The MIGWAS method first integrates the results of genome-wide association studies of cell- and tissue-type specific expression profiles of miRNAs. Afterwards, it employs miRNA-gene target prediction algorithms to pinpoint the tissue/cell-specific contribution of individual miRNAs to particular diseases and identify miRNAs that might serve as potential biomarkers. With this method, the study has discovered 39 miRNA-target gene pairs consisting of 37 individual genes and 16 distinctive miRNAs. Additionally, it has shown the enrichment of miRNA-target genes in 25 separate examined tissues without accounting for tissue-specific miRNA profiles. Of all the identified tissues, the highest enrichment was found in the keratinised cells of the oral mucosa of the gastrointestinal tract [387]. This is a somewhat expected result, as there is a mounting body of evidence of a bidirectional, proinflammatory relationship between MS and the gut microbiome. The pathologically composed gut microbiome of MS patients was shown to induce a proinflammatory state and, vice versa, the autoreactive immune system of the patient has been observed to shape the gut microbiome [388,389,390]. The vast majority of the biomarker candidate miRNA-target gene pairs identified by the study are already known to be involved in the activation and signalling apparatus of the immune system. The genes reported are implicated in T-cell activation, class II HLA expression, TGF-ß signalling, proteosome function and degradation targeting, neuronal differentiation and synaptic transmission, also protein folding and homeostasis upkeep of the endoplasmic reticulum. Of the 16 reported miRNAs, five have already been associated with MS; mir-3605 was identified as a candidate biomarker for pedMS by the authors, a result verified by subsequent confirmation studies [391,392]. Interestingly, only one, the TVP23B gene, was identified by the miR-SNP analysis as being associated with pedMS. Its exact role in the pathogenesis of MS is unclear, as this gene has been previously linked to diabetic retinopathy, but not MS [393]. Statistical overrepresentation tests conducted on the miR-SNP analysis have shown that a total of 30 genes in 5 crucial signalling pathways are heavily dysbalanced in pedMS patients. The analysis has shown the aberrant regulation of 5 genes in the histamine H_1_ receptor, five genes in the MHC protein complex, five genes present on the inner part of the ER lumen, six genes in the 5-HT_2_ type receptor-mediated signalling pathway, and nine genes in the interferon-gamma signalling pathways. Furthermore, as expected, pedMS patients were shown to have more copies of the HLA-DRB1*15:01 allele than controls [387].

The results of this large scale study strongly suggest that a disbalance in and subsequent faulty regulation by the miRNA system is a significant contributing factor to the development of pediatric MS.

The vast amount of research that in recent years has been directed at the miRNA system and its role in MS pathology has resulted in significant progress. Detailed profiles of disease-related cells and tissue types and a plethora of aberrantly regulated miRNAs in the plasma, CSF, serum, NAWM and CNS lesions of MS patients have been identified recently, some of which have the potential to become markers for diagnosis and/or progression [394,395,396,397,398,399,400,401,402,403].

As mentioned previously in this section, the binding of a miRNA to its target mRNA causes the degradation of the mRNA molecule in the case of full complementarity or just the inhibition of its translation in the case of incomplete complementarity. Due to redundancy and pleiotropy, the miRNA system is thought to regulate the expression of more than 60% of the human protein-coding genes [358]. Even though several research groups have identified various miRNAs (more than 700 miRNAs are currently recognised to be dysbalanced in MS) associated with different aspects of MS pathology, there is, unfortunately, significant heterogeneity among the results of the studies. Accordingly, a meta-analysis dealing with the matter has found staggeringly low reproducibility across studies; less than 10% of the identified miRNAs were found to be imbalanced in the same direction by at least three independent reports [402,403,404]. Currently, the single most reliable diagnostic marker for MS is serum miR-145 with a sensitivity close to 90% [402]. A pair of studies [375,405] attempted to resolve this problem by trying to link previously reported miRNAs to disease activity characterised by MRI lesion activity. A total of 23 miRNAs were identified this way, with consistent expressional changes and a strong correlation to lesion burden and activity. Another large scale MRI-based study [406] has identified three other miRNAs (all playing a role in the upkeep of the blood-brain barrier) as potential differentiators and markers of disease phenotype and progression.

Due to this observed heterogeneity in results, several obstacles have yet to be overcome for miRNAs to become valid, easily comparable and reproducible biomarkers in MS. One of the first challenges is pinpointing the exact members among the severely dysbalanced miRNAs that are not just bystanders but also key players in MS pathogenesis and progression. Another chief problem is the low reproducibility of the studies in the literature. The reason for this is multifactorial. First, miRNA expression is influenced by several factors such as age, disease course, sex, and prior or concomitant immunmodulant and steroid treatment. Second, there is significant heterogeneity in the research protocols used, patient selection, the techniques used to isolate and sequence miRNA expression from different kinds of tissues/fluids. Furthermore, various methodologies are used in analysing the gathered data. At the time of writing this article, there is no standardised method for miRNA profiling or data analysis or patient selection to avoid known confounding factors. This needs to be addressed to overcome the current inter-study heterogeneity and low reproducibility issues. Another obstacle is the precise evaluation of miRNA expression in different cell types. Most of the published reports used bulk RNA sequencing to measure miRNA levels in the blood (plasma or serum), the CNS or other tissues of MS patients. Therefore, evaluating a specific miRNA’s expression level in a single given cell type is extremely difficult. Some research groups have overcome this barrier and have been able to clarify the individual roles of specific miRNAs in peripheral cell types (mostly in PBMCs and lymphocytes). Unfortunately, accomplishing the same in the resident cells of the CNS (microglia, neurons, oligodendrocytes and astrocytes), which are implicated in MS pathogenesis, lesion activity and repair, still remains a challenge [372,407]. In order to step forward in the future, it will be crucial to identify miRNAs that are dysregulated in microglia, neurons, oligodendrocytes and astrocytes, specifically in MS pathology, particularly when it comes to identifying the most effective targets for therapeutic intervention. Should these challenges be overcome, miRNAs have the potential to become extremely useful diagnostic and progression markers; furthermore, they may also give us insight into the pathology of MS as well.

### 2.10. CircularRNA (circRNA)

Circular RNAs (circRNA) were first described some 45 years ago by Sanger et al. [408]. Still, it was only recently that their regulatory function and biological significance had been confirmed in both physiological and pathological processes. As mentioned in the previous section on miRNAs, the relative failure in the search for the genetic predisposition to MS has shifted the spotlight towards epigenetic factors. This led to the re-discovery of miRNAs and sparked significant interest in their role in autoimmune diseases such as MS [396,409,410,411,412]. As the network regulated by miRNAs began to unfold, their redundancy and ability to regulate several different targets was confirmed by several subsequent studies. It became clear that there has to be a yet unknown regulatory mechanism keeping miRNAs in check. Accumulating evidence suggests that one role of circRNAs might be just this; one of their chief functions may be the miRNA system’s posttranscriptional regulation.

Similarly to miRNAs, CircRNAs are a species of extremely stable endogenous RNAs found in the blood and other biofluids. Due to the lack of conventional RNA tails, circRNAs are resistant to the RNA endonucleases, resulting in their half-life being well over 48 h [413]. CircRNAs are independently regulated from that of their host genes [414]. They are the products of alternative splicing via four different pathways during which a downstream 5′ splice donor site is joined to a 3′ upstream splice acceptor site [415,416,417]. Circular RNAs seem to be a complex group of molecules reflecting their diverse biological role and targets. Based on their origin, three main subtypes have been identified so far; intronic (ciRNA), exonic (ecircRNA) and exon-intron containing circRNAs (EIcircRNA). Most of the known circRNAs are of exonic origin and located in the cytoplasm; meanwhile, ciRNAs are mainly localised in the nucleus and constitute a much smaller fraction of circRNAs. EIcircRNAs are located in the nucleus as well, and they are suggested to play a role in the promotion of transcription [418,419]. The circRNA system is highly redundant; a single circRNA can contain more than one binding site for an individual miRNA molecule; concomitantly, the same circRNA might bind several different miRNAs simultaneously. Due to this attribute, circRNAs are referred to as intrinsic "sponges” for miRNAs [420,421,422]. When a circRNA captures a miRNA, the bound miRNA’s target messenger RNA is freed from suppression, and transcription may ensue. This way, circRNAs essentially act as posttranslational regulators for the miRNA system. In addition, circRNAs were shown to bind and sequester RNA binding proteins, thus regulating translation and protein production in a miRNA independent manner [415]. Additionally, circRNAs are documented to have impeccable complementarity to linear miRNA species [415], further expanding their numerous yet unexplored function in biological processes.

Interestingly, inverse correlation has been found between a cell type’s proliferation rate and circRNA concentration. Rapidly proliferating cell lines are documented to contain less circRNA than cell types with a low proliferation rate [423]. This is in line with the observation of the abundance of circRNAs in the brain compared to other tissues in humans [424]. In recent years, circRNAs have been implicated in various immune processes, and immune-mediated disorders also are key players in several diseases affecting the CNS, including MS [425,426,427,428,429,430,431,432]. A few years ago, the first circRNAs to be dysregulated in MS patients were discovered [429,430]. The identified circRNAs were shown to be connected to various genes (Gasdermin B, MALAT1, IL-7 receptor, SP140) already linked to the pathogenesis of MS. Furthermore, they were also discovered to have a different pattern of expression in relapse and remission and female and male MS patients [432,433,434,435,436,437,438,439]. Using a genome-wide association technique [440], the same group found significant enrichment of non-coding elements in the genomic regions harbouring known MS-associated SNPs. A total of 482 circRNAs were found in the areas of interest vs a mean of 194 ± 65 in the random sets. This way, a total of 18 circRNAs were identified (of which two were novel) as derived from MS-associated genes by the RNA sequencing of two cell lines (SH-SY5Y and Jurkat, both representing tissues relevant for MS). Furthermore, a circRNA (hsa_circ_0043813) from the STAT3 gene (a transcription factor responsible for the polarization of the immune response toward Th17 and thus the development of inflammation) was confirmed to be modulated by three genotypes at the disease-associated SNP. This was later corroborated by the findings of another study, which documented circRNAs affecting the STAT3 pathway to be differently expressed in RRMS patients compared to controls [441]. The downregulation of two additional circRNAs (circ_0005402 and circ_0035560) was reported in MS patients [428]. These circRNAs are located in the ANXA2 gene, which most recently was associated with blood-brain barrier dysfunction in a mouse model of MS [442]. In addition to circRNAs being implicated in the pathogenesis of MS, they have also been linked to disease activity. Accordingly, three (hsa_circRNA_101348, hsa_circRNA_102611, and hsa_circRNA_104361) circRNAs targeting 15 miRNAs and three additional protein-coding RNAs (of which 2 AK2 and IKZF3 are known to be involved in B-cell function) were measured to be overexpressed during a relapse [443]. The disease activity-related expression of various circRNAs was corroborated by a subsequent study, which has additionally described the sex-dependent increase of circRNA expression in MS, and furthermore has validated and proposed six additional circRNAs as potential biomarkers [431]. The circRNA profile of an individual might not just be an excellent biomarker to monitor relapse activity, but based on recent research, it may be used to differentiate between MS subtypes. Iparraguirre et al. [444] have found that healthy controls and patients with SP and RR MS have fundamentally different circRNA profiles. They also demonstrated that miRNA "sponging” might not be circRNAs primary function in extracellular vesicles and leukocytes despite prior knowledge. Moreover, the expression of some circRNAs (hsa_circ_0000478 and hsa_circ_0116639) were found to correlate with disease severity and the presence of the anti-myelin lipid-specific oligoclonal IgM bands in the CSF of MS patients [445].

All of the continuously accumulating data suggest that various non-coding RNA species and the so-called "competing endogenous RNA network” (the detailed reporting of which is unfortunately well beyond the scope of this article, for very extensive literature and a review on the matter see [446,447,448,449]) might contribute to MS pathogenesis via several already known and yet to be discovered pathways and mechanisms. Circular RNAs are prominent members of this family onto whom intense research has been focused in recent years. Owing to the findings of this intensive research, the circRNA profiling of MS patients may become a reliable tool to predict future disease activity and accurately identify the disease phenotype at diagnosis. Furthermore, the changes in one’s non-coding RNA profile may become a viable method to monitor disease activity in the near future.

## 3. Conclusions

MS is often called the lupus of neurology due to the heterogeneity of symptoms it can present with. This diversity is also true for the distinctive pathological processes [5,450] leading to the development of the disease. Due to the debilitating and irreversible damage MS can cause if left unchecked, it is crucial to diagnose it in the early stages and to commence adequate treatment as soon as possible [451]. The current era in MS, with rapidly expanding therapeutic options, has raised the demand for prognostic and diagnostic biomarkers and accurate, accessible, validated and minimally invasive markers to closely monitor disease activity, disability progression, and therapeutic response. The aim of treating MS patients currently is fundamentally different from that seen decades ago when the slowing of progression was the only achievable aspiration. The current objective is to offer patients completely personalised treatment and a complete halt of the disease, thus preventing any further injury and, if possible, reverse some of the already sustained damage to the CNS [452,453,454,455,456,457,458]. The disease-modifying treatments used at present are immunomodulatory or immunosuppressive in nature. Currently, the only option for monitoring their therapeutic efficacy in everyday practice is clinical (i.e., the presence or absence of relapses or the presence or lack of EDSS progression in progressive disease forms) and/or MRI measures (i.e., new or equivocally enlarging lesions, number of black holes, contrast enhancement and, if available, total brain/grey matter atrophy). The problem with these methods in conventional MS care is their inability to forecast disease activity, and they might not capture subclinical processes [185], e.g., the development of psychopathological symptoms [459], cognitive dysfunction [460,461,462], attention network deficits [463] and subtle neurodegeneration. The soluble biomarkers found in the CSF and/or blood included in this review may eliminate this problem. They can provide the physician with invaluable information on the actually and subclinically ongoing inflammation and neurodegeneration in the CNS of MS patients. These biomarkers are of variable nature (cytokines, chemokines, RNA species etc.). On the one hand, they reflect the diversity of the immune system’s involvement in MS pathogenesis and disease progression. On the other hand, they can be used to monitor fundamentally different aspects of the disease. Despite the great progress seen in the past couple of years, there are still several challenges that are yet to be overcome for these biomarkers to become reliable pillars of routine MS care. First of all, none of these markers is specific, neither for MS nor for the CNS (except for neurofilaments, which are specific for neural damage). As such, other concomitant diseases (e.g., infections, CNS trauma, stroke, inflammatory or non-inflammatory neurological disease) that affect the immune system and/or damage the CNS may influence the kinetics and concentrations of these markers. Furthermore, the currently used DMTs have different mechanisms of action and molecular targets; therefore, not every drug will influence the same biomarkers and might affect the ones they do influence with different power. Moreover, a given marker’s CSF and blood levels by a given DMT might be influenced with different power, making an accurate interpretation even harder. Yet another limitation of many of these inflammatory markers is their sensitivity to degradation by various proteases; therefore, the samples’ adequate and rapid pre-analytical handling and storage is crucial for getting valid and reproducible results. However, the biggest hurdle is that even though there is rapidly accumulating data regarding each respective biomarker included in this review, unfortunately, most of the evidence is based on studies of limited size, the results of which are sometimes contradictory. Hopefully, this will change shortly, as owing to recent advances in analytical technology, biomarkers that were previously only detectable in the CSF have now become measurable in blood. This makes repeat lumbar puncture futile and allows for repeat sampling and the less invasive surveillance of MS, thus making the recruitment of bigger study cohorts much simpler. A prime example of this transition from CSF to blood is the case of NfL, which, thanks to the development of SiMoA, has become the prime focus of biomarker research in MS, so much so that it is included in more and more RCTs as a non-primary endpoint.

Based on current trends, the extraordinarily diverse and intertwined processes that constitute the pathophysiology of MS and available research data, it is doubtful that a "holy grail” of biomarkers exists for MS. A much more plausible scenario is that the biomarkers above are combined as a panel and/or a patient’s repeated biomarker profiling is going to be a specific and sensitive enough measure to complement the currently used clinical and MRI parameters in monitoring disease activity, planning therapeutical decisions and truly making medicine more personalised. Despite significant advances in the past couple of years, there is still much to do for this to become a reality. Further studies with larger cohorts and more uniform methodologies are needed to validate current and new biomarkers to become solid enough to be incorporated into diagnostic criteria and become widespread enough to become part of routine MS care. Indeed, the next few years in biomarker research will be extremely exciting, as it is an exceptionally vibrant scientific area and great breakthroughs are much awaited.

## 4. Limitations

The biggest limitation of our review lies in its narrative nature. In contrast to systematic reviews, the lack of stringent inclusion criteria and methodical analysis in narrative reviews makes them futile to be used as solid scientific evidence. On the other hand, the same loose criteria allows for the critical analysis of a much larger spectrum of the literature and can provide readers with up-to-date knowledge about a specific topic or a theme, not just a specific question, which was the authors’ intention. Nonetheless, due to the narrative approach used in the present review, careful conclusions must be drawn.

## Data Availability

Not applicable.
